# Revisiting the Role of Toxoplasma gondii ERK7 in the Maintenance and Stability of the Apical Complex

**DOI:** 10.1128/mBio.02057-21

**Published:** 2021-10-05

**Authors:** Nicolas Dos Santos Pacheco, Nicolò Tosetti, Aarti Krishnan, Romuald Haase, Bohumil Maco, Catherine Suarez, Bingjian Ren, Dominique Soldati-Favre

**Affiliations:** a Department of Microbiology and Molecular Medicine, Faculty of Medicine, University of Geneva, Geneva, Switzerland; University of Pittsburgh

**Keywords:** Apicomplexa, *Toxoplasma gondii*, conoid, apical cap, subpellicular microtubules, comparative proteomics, egress, motility, invasion, microneme secretion, extracellular signal-regulated kinase, microtubule, apicomplexan parasites, host cell invasion

## Abstract

Toxoplasma gondii extracellular signal-regulated kinase 7 (ERK7) is known to contribute to the integrity of the apical complex and to participate in the final step of conoid biogenesis. In the absence of ERK7, mature parasites lose their conoid complex and are unable to glide, invade, or egress from host cells. In contrast to a previous report, we show here that the depletion of ERK7 phenocopies the depletion of the apical cap protein AC9 or AC10. The absence of ERK7 leads to the loss of the apical polar ring (APR), the disorganization of the basket of subpellicular microtubules (SPMTs), and a severe impairment in microneme secretion. Ultrastructure expansion microscopy (U-ExM), coupled to *N*-hydroxysuccinimide ester (NHS-ester) staining on intracellular parasites, offers an unprecedented level of resolution and highlights the disorganization of the rhoptries as well as the dilated plasma membrane at the apical pole in the absence of ERK7. Comparative proteomics analysis of wild-type and ERK7-depleted parasites confirmed the disappearance of known apical complex proteins, including markers of the apical polar ring and a new apical cap named AC11. Concomitantly, the absence of ERK7 led to an accumulation of microneme proteins, resulting from the defect in the exocytosis of the organelles. AC9-depleted parasites were included as controls and exhibited an increase in inner membrane complex proteins, with two new proteins assigned to this compartment, namely, IMC33 and IMC34.

## INTRODUCTION

The Apicomplexa phylum is defined by the so-called apical complex, a structure that harbors unique secretory organelles, termed rhoptries and micronemes, as well as membranous and cytoskeletal elements that critically contribute to parasite dissemination. The overall cytoskeleton is made of three interacting layers, conferring structure and stability to the parasite: the inner membrane complex (IMC), formed by a patchwork of flattened vesicles; the subpellicular network (SPN), composed of intermediate filament-like proteins termed alveolins; and the subpellicular microtubules (SPMTs). The most apical region of the IMC, called the apical cap, is formed by a single vesicle and was shown to participate in apical complex stability (1–3). Members of the coccidian subgroup possess an additional open-barrel-shaped structure made of unique fibers of tubulin polymers termed the conoid (4), with two preconoidal rings (PCRs) at the top and two short intraconoidal microtubules on the inside (5). The conoid lies within the apical polar ring (APR), which serves as the microtubule-organizing center (MTOC) for the spiraling SPMTs. Apical cap protein 9 (AC9) and AC10 were previously shown to be SPN-resident proteins whose depletion resulted in the loss of the conoid and APR as well as the disorganization of the SPMTs at the apical pole (1, 2). The conoid is the site of convergence for calcium- and lipid-mediated signaling cascades that coordinate microneme secretion and actin polymerization and is also a site for the glideosome, the molecular machinery powering parasite gliding motility (6). Strikingly, AC9- and AC10-depleted parasites were severely impaired in microneme secretion and consequently were unable to move, invade, or egress from host cells. We postulated that the loss of the conoid and SPMT disorganization could result from the disappearance of the APR. Concordantly, another study reported that the double knockout of two APR proteins (APR1 and KinA) led to the fragmentation of the APR, partial detachment of the conoid, and reduced microneme exocytosis (7). In contrast, the depletion of the mitogen-activated protein kinase (MAPK) extracellular signal-regulated kinase 7 (ERK7) reportedly caused the loss of conoid in mature parasites without seemingly affecting microneme secretion or APR integrity (3). Moreover, AC9 was assigned a dual role in localizing ERK7 at the apical cap and in regulating its kinase activity and substrate specificity (2). Another protein, the conoidal ankyrin repeat-containing protein (CPH1), was also reported to contribute to conoid integrity in extracellular parasites. CPH1-depleted parasites harbored shortened, partially collapsed conoids, again without seemingly impacting microneme secretion (8).

Here, we revisited the role of ERK7 in microneme secretion. The depletion of ERK7 phenocopies the depletion of AC9 or AC10, leading to not only the loss of the conoid but also the disappearance of the APR and a complete block of induced microneme secretion. A comparative proteomics analysis of wild-type and ERK7- or AC9-depleted parasites highlighted the loss of known as well as novel candidate proteins composing the apical complex. Conversely, the accumulation of microneme proteins reflected the severe defect in the exocytosis of these secretory organelles in the absence of ERK7.

## RESULTS

### ERK7 is essential for the stability of the conoid complex and the organization of the subpellicular microtubules in mature parasites.

Three mitogen-activated protein kinases (MAPKs) are encoded in the genome of Toxoplasma gondii and conserved across the superphylum of the Alveolata, except for TgMAPK-like 1, which is missing in the Haemosporida and Piroplasmida orders (9, 10); their respective localizations and functions in T. gondii and *Plasmodium* spp. are summarized in Fig. S1A in the supplemental material. Briefly, both TgMAPK-like 1 and TgMAPK2 are involved in different steps of parasite replication (11, 12), while TgERK7 is involved in conoid biogenesis, thus hampering parasite invasion, egress, and dissemination (3). The *Plasmodium* species orthologues of TgERK7 (Plasmodium falciparum/P. berghei MAP-1 [Pf/PbMAP-1]) and TgMAPK2 (Pf/PbMAP-2) are dispensable in the asexual blood stage, with only Pf/PbMAP-2 being required for male gametogenesis (13–16).

10.1128/mBio.02057-21.1 FIG S1 (A) Mitogen-activated protein kinases (MAPKs) present in T. gondii and *Plasmodium* species 1 (11), 2 (3), 3 (48), 4 (49), 5 (50), 6 (13), 7 (12), 8 (14), 9 (15), and 10 (16). (B) ERK7 was C-terminally tagged with the mAID-HA construct as shown by integration PCRs. (C) The ERK7-mAID-HA construct was tightly downregulated upon the addition of auxin (IAA). (D) ERK7 localized at the apical cap of both mature and daughter cells. Like AC9 and AC10, ERK7 is among the earliest markers appearing during endodyogeny, before the daughter cytoskeletal basket. Bars = 2 μm. (E) ERK7-depleted parasites failed to form lysis plaques after 7 days. TIR1 represents the parental strain. (F) Representative wide-field pictures (maximum projection) of extracellular ERK7-mAID-HA parasites treated or not with IAA, as seen after a typical U-ExM experiment. Treated parasites clearly displayed abnormal SPMT arrangements, missing conoids, and enlarged apical poles (seen only when well oriented). Bars = 20 μm. (G) The localization of another apical cap protein (ISP1) was not majorly impacted by ERK7 degradation; nevertheless, the apical region of some parasites per vacuole is clearly enlarged, and some show no or reduced ISP1 signals (arrowhead). Bars = 2 μm. Download FIG S1, EPS file, 2.6 MB. Copyright © 2021 Dos Santos Pacheco et al. 2021 Dos Santos Pacheco et al. https://creativecommons.org/licenses/by/4.0/ This content is distributed under the terms of the Creative Commons Attribution 4.0 International license.

**FIG 1:**
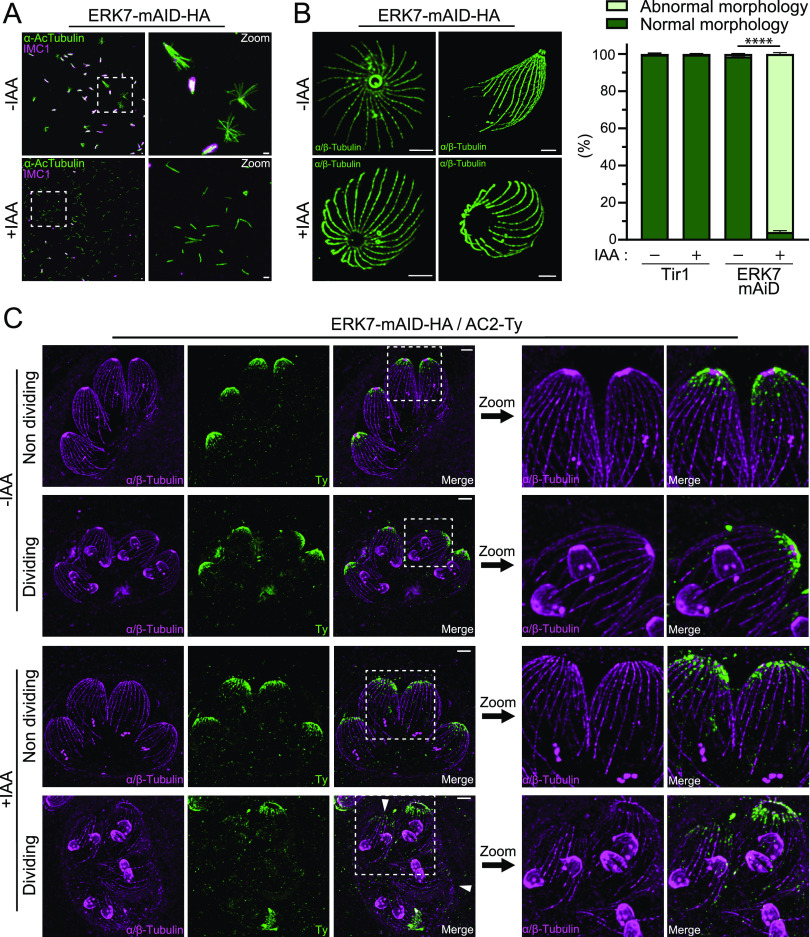
ERK7 depletion caused major cytoskeletal defects at the apical pole. (A) Extracellular parasites were extracted with deoxycholate (DOC) and placed on gelatin-coated coverslips. Depletion of ERK7 caused the collapse of the microtubular cytoskeleton as only single microtubules can be visualized by IFAs with anti-acetylated tubulin (AcTubulin) antibody. Bars = 2 μm. (B) Ultrastructure expansion microscopy (U-ExM) highlighted the absence of the APR and consequently an enlargement of the apical pole and disorganization of the cytoskeleton in ERK7-depleted parasites. On the right are quantifications of abnormal parasites (missing a conoid and/or with an enlarged apical pole and/or disorganized microtubules) versus normal parasites by U-ExM. For the four conditions presented, 200 parasites were counted for each of the three independent biological replicates. Bars = 2 μm. ****, *P* < 0.0001. (C) U-ExM was applied under intracellular conditions with AC2 tagged in the ERK7-inducible strain. Upon the addition of IAA, the conoid is missing exclusively in the mother cell, and in some severe cases of SPMT disorganization, AC2 staining disappears from the apical cap as well (arrowhead). Bars = 2 μm.

TgERK7 was carboxy-terminally tagged with a mini auxin-induced degron cassette and hemagglutinin epitope tags (ERK7-mAID-HA) at the endogenous locus and checked for integration by PCR analysis of genomic DNA (Fig. S1B). The protein was shown to be tightly downregulated upon the addition of auxin (indole acetic acid [IAA]) (Fig. S1C) and localized at the apical cap of both mature and daughter cells (Fig. S1D). As previously reported, parasites depleted of ERK7 failed to form lysis plaques in a monolayer of human foreskin fibroblasts (HFFs) after 7 days of IAA treatment (Fig. S1E). Given the close functional relationship reported between AC9 and ERK7, cytoskeletal integrity was checked by deoxycholate (DOC) extraction. As observed in the absence of AC9 or AC10, the cytoskeleton of parasites depleted of ERK7 dramatically disassembled (Fig. 1A). In addition to the loss of conoid, ultrastructure expansion microscopy (U-ExM) images of extracellular parasites clearly highlight the disorganization of the SPMTs (Fig. S1F). Also, the APR seems to be lost upon the depletion of ERK7, leading to a wider apical opening (Fig. 1B). This potentially explains the detached SPMTs visible by DOC extraction and the disorganized SPMT basket. U-ExM was performed on intracellular parasites for the first time, using the ERK7-mAID-HA strain further modified by Ty epitope tagging of AC2, a marker of the alveolin network (ERK7-mAID-HA/AC2-Ty). As previously shown with AC9-depleted parasites, the absence of ERK7 led to the loss of the conoid in mature parasites, while the nascent daughter cells still possessed an intact apical complex (Fig. 1C). Overall, AC2 was not dramatically affected by ERK7 depletion; however, occasionally, AC2 either was not present apically or was markedly reduced in some parasites (Fig. 1C). The same observation was made with another apical cap protein, ISP1 (Fig. S1G).

### The apical polar ring is lost in mature ERK7-depleted parasites.

**FIG 2:**
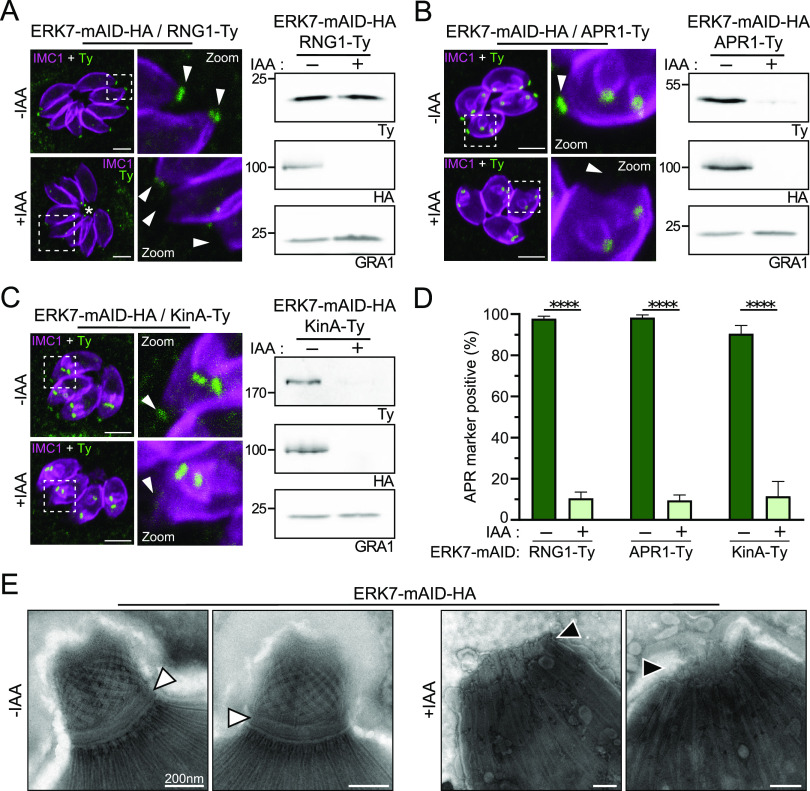
Loss of APR and APR markers in ERK7-depleted parasites. (A) RNG1, a late marker of parasite division, failed to be incorporated into most of the parasite APRs. An RNG1 signal could be detected in the parasite cytoplasm/residual body (asterisk). Western blot analysis confirmed that RNG1 is not degraded. Bars = 2 μm. (B and C) Two additional APR markers, APR1 and KinA, are incorporated early during daughter cell formation and are exclusively lost in mature parasites following the depletion of ERK7. Western blot analysis of extracellular parasites treated with IAA showed that both proteins are degraded. Arrowheads indicate the mother cell apex. Bars = 2 μm. (D) Quantification of vacuoles in which a parasite displays a normal apical RNG1, APR1, or KinA marker. For the six conditions presented, 200 vacuoles were counted for each of the three independent biological replicates. ****, *P* < 0.0001. (E) EM pictures showing that ERK7 depletion caused the physical loss of the APR and an enlargement of the apical pole of extracellular parasites, in good agreement with the IFA using the RNG1, APR1, and KinA markers. White arrowheads indicate a normal APR; black arrowheads indicate a missing APR. Bars = 200 nm.

To confirm the loss of the APR, three APR markers were epitope tagged in the ERK7-mAID-HA strain, namely, RNG1, APR1, and KinA (7, 17). Upon ERK7 depletion, RNG1, a very late maker of parasite division, was not visible at the APR of mature parasites (Fig. 2A and D; Fig. S2A), whereas the protein was still detectable by Western blotting (Fig. 2A). Remarkably, the remaining RNG1 signal is mislocalized in the absence of ERK7, accumulating mainly at the posterior pole of the parasites (Fig. 2A; Fig. S2A). The two other APR markers were lost exclusively in the mature parasite but present in nascent daughter cells, confirming the role of ERK7 only during the late stages of parasite division (Fig. 2B and C; Fig. S2B and C). In contrast to RNG1, both APR1 and KinA are degraded based on Western blot analysis (Fig. 2B and C). In addition to an immunofluorescence assay (IFA), we used electron microscopy (EM) on extracellular parasites to assess the disappearance of the APR. A wider opening at the apical pole and the loss of the APR were evident in ERK7-depleted parasites (Fig. 2E; Fig. S2D).

10.1128/mBio.02057-21.2 FIG S2 (A to C) Raw pictures and quantification of RNG1 (A), APR1 (B), and KinA (C) in the ERK7-inducible strain. Graphs from these raw data are displayed in Fig. 2D. Bars = 2 μm. (D) Gallery of additional EM images showing that ERK7 depletion caused the physical loss of the APR and an enlargement of the apical pole of extracellular parasites, as seen in Fig. 2E. White arrowheads indicate normal APRs; black arrowheads indicate missing APRs. Bars = 200 nm. Download FIG S2, EPS file, 2.8 MB. Copyright © 2021 Dos Santos Pacheco et al. 2021 Dos Santos Pacheco et al. https://creativecommons.org/licenses/by/4.0/ This content is distributed under the terms of the Creative Commons Attribution 4.0 International license.

### U-ExM coupled to NHS-ester staining highlights several morphological defects in parasites lacking ERK7.

**FIG 3:**
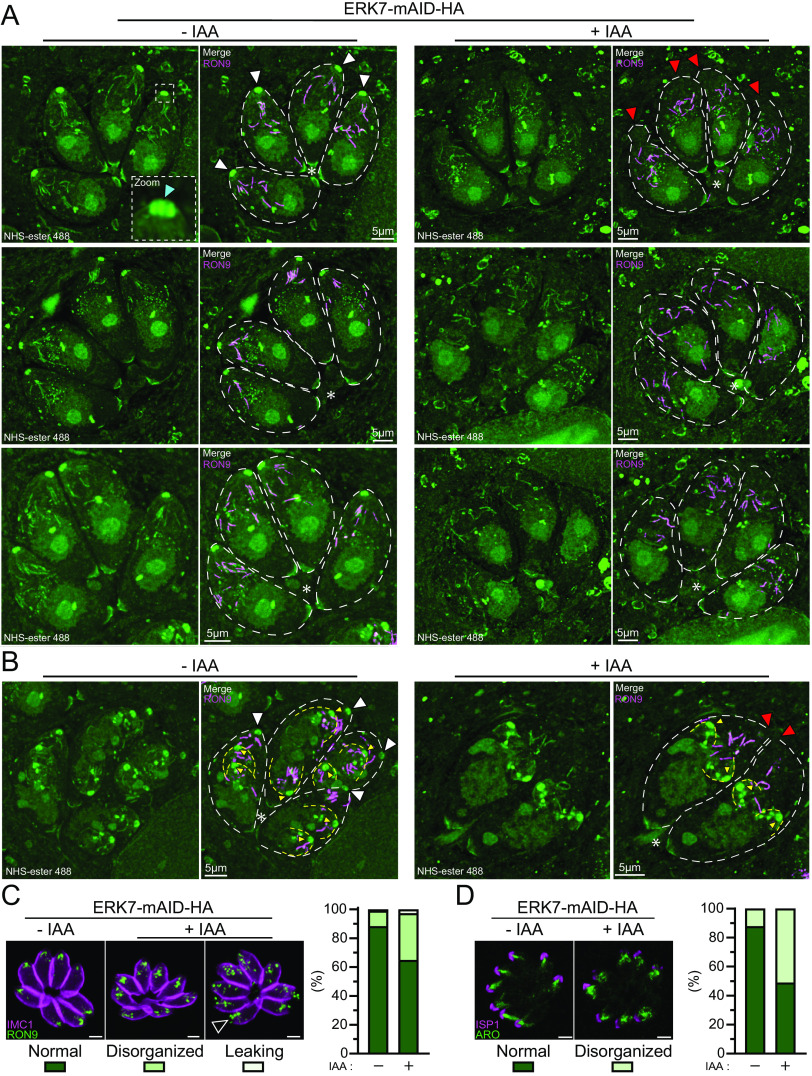
NHS-ester coupled with U-ExM highlights conoid loss, rhoptry disorganization, and plasma membrane slackness. (A) Maximum-projection pictures of ERK7-untreated and -treated parasites. The top raw images are extracted from Movies S1 and S3 in the supplemental material (sometimes rotated to have the majority of the parasite apical pole facing up). All images are presented with NHS-ester staining only (left) and with RON9 immunostaining and some annotations (right). In the top raw images, white arrowheads indicate mother cell conoids (light blue arrowhead, intraconoidal microtubules), and red arrowheads indicate the deformation of the apical plasma membrane and the absence of the mother cell conoid. Dotted lines follow the shape of mature parasites (white). Asterisks indicate the basal pole for each vacuole. (B) Same as panel A, with parasites in division (from Movies S2 and S4). Yellow dotted lines follow the shape of the forming daughter cells. (C and D) Regular immunofluorescence highlights rhoptry disorganization using RON9 and ARO antibodies. Quantification of ∼100 vacuoles on a single biological replicate is shown solely for information purposes. Bars = 2 μm.

Next, to gain further insight into the morphology of the ERK7-depleted parasites, we coupled U-ExM newly adapted to T. gondii (1) with the fluorescent dye *N*-hydroxysuccinimide ester (NHS-ester) able to bind to primary amines of proteins. Remarkably, this combination allows the observation of a wide range of anatomical features, some of which were so far visible only by EM, and in a direct and unbiased way (18). This technique allowed the visualization of a wide spectrum of recognizable ultrastructures (Movies S1 and S2), with a nonexhaustive list of such features presented in individual stacks (Fig. S3A to F). As expected, in ERK7-depleted parasites, the conoid was clearly absent from mature parasites while still being present in forming daughter cells (Fig. 3A and B; Movies S3 and S4). Interestingly, when the rhoptries were stained by NHS-ester and anti-RON9 antibodies, the so-called “neck” seemingly did not extend all the way up to the conoid (Fig. 3A). Also, the rhoptry bulbs were very poorly stained by NHS-ester. Finally, and as previously observed by EM for AC9-depleted parasites (1), the plasma membrane of some parasites was “dilated” at the apical pole in the absence of ERK7 (Fig. 3A).

10.1128/mBio.02057-21.3 FIG S3 Individual stacks from Movies S1, S3, and S4 in the supplemental material highlighting the broad spectrum of NHS-ester staining for ultrastructural elements and organelles. On the top left is the stack number. (A) Ring structures observed on the parasite surface (from Movie S1). (B) Apicoplast (from Movie S1). (C) Rhoptry neck (from Movie S1). (D) Post-Golgi vesicles (from Movie S3). (E) Apical microneme collars just below the conoid (from Movie S1). (F) Centrocone (white arrowhead) and centromere (yellow arrowhead) during parasite division (from Movie S4). Bars = 5 μm. (G and H) Gallery of additional immunofluorescence images displaying the rhoptry disorganization upon the depletion of ERK7. Anti-RON9 detects the rhoptry neck, while anti-ARO highlights the whole surface of the organelle. Bars = 2 μm. Download FIG S3, EPS file, 2.5 MB. Copyright © 2021 Dos Santos Pacheco et al. 2021 Dos Santos Pacheco et al. https://creativecommons.org/licenses/by/4.0/ This content is distributed under the terms of the Creative Commons Attribution 4.0 International license.

10.1128/mBio.02057-21.7 MOVIE S1 ERK7-untreated parasites (−IAA), nondividing cells. Download Movie S1, MOV file, 2.9 MB. Copyright © 2021 Dos Santos Pacheco et al. 2021 Dos Santos Pacheco et al. https://creativecommons.org/licenses/by/4.0/ This content is distributed under the terms of the Creative Commons Attribution 4.0 International license.

10.1128/mBio.02057-21.8 MOVIE S2 ERK7-untreated parasites (−IAA), dividing cells. Download Movie S2, MOV file, 4.0 MB. Copyright © 2021 Dos Santos Pacheco et al. 2021 Dos Santos Pacheco et al. https://creativecommons.org/licenses/by/4.0/ This content is distributed under the terms of the Creative Commons Attribution 4.0 International license.

10.1128/mBio.02057-21.9 MOVIE S3 ERK7-treated parasites (+IAA), nondividing cells. Download Movie S3, MOV file, 8.5 MB. Copyright © 2021 Dos Santos Pacheco et al. 2021 Dos Santos Pacheco et al. https://creativecommons.org/licenses/by/4.0/ This content is distributed under the terms of the Creative Commons Attribution 4.0 International license.

10.1128/mBio.02057-21.10 MOVIE S4 ERK7-treated parasites (+IAA), dividing cells. Download Movie S4, MOV file, 5.1 MB. Copyright © 2021 Dos Santos Pacheco et al. 2021 Dos Santos Pacheco et al. https://creativecommons.org/licenses/by/4.0/ This content is distributed under the terms of the Creative Commons Attribution 4.0 International license.

### ERK7-depleted parasites are defective in induced microneme secretion.

**FIG 4:**
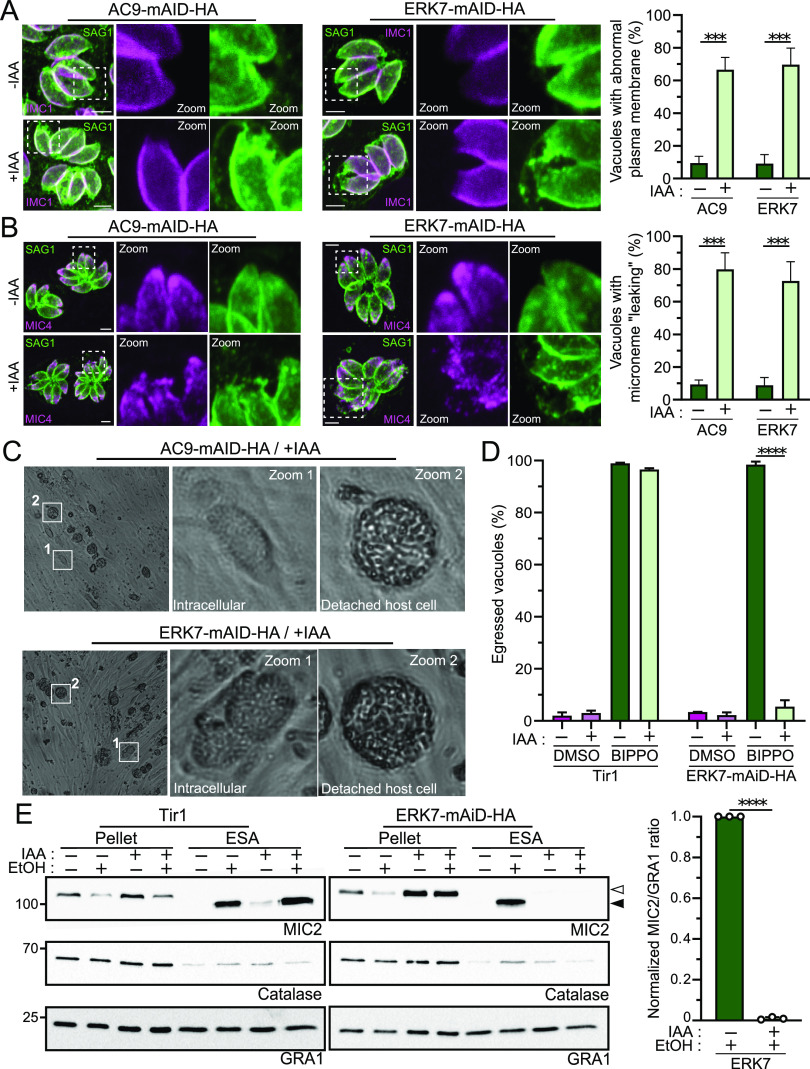
ERK7-depleted parasites are defective in PM integrity and microneme secretion. (A and B) For both AC9 and ERK7 depletion, some parasites per vacuole showed a defect in plasma membrane integrity at the apical tip (A) and leaking of micronemes (B), highlighted by SAG1 and MIC4 staining, respectively. For the four conditions presented, 200 vacuoles were counted for each of the three independent biological replicates. Bars = 2μm. ***, *P* < 0.001. (C) Large bright-field images of AC9 and EKR7 parasites grown for 48 h in the presence of IAA. Depleted parasites showed no defects in intracellular growth (intracellular); however, parasites remained trapped inside the floating host cell remnant (detached host cells), suggesting microneme secretion impairment. (D) ERK7 displayed a severe defect in a “standard” induced-egress assay. For each condition presented, 200 vacuoles or PVM remnants were counted for each of three independent biological replicates. ****, *P* < 0.0001. DMSO, dimethyl sulfoxide. (E) Depletion of ERK7 caused a dramatic block in microneme secretion when the parasites were stimulated with ethanol (EtOH). Anti-MIC2 antibodies were used for secretion (white arrow, full-length MIC2; black arrow, secreted MIC2), anti-catalase (CAT) to assess parasite lysis, and anti-dense granule 1 (GRA1) for constitutive secretion; both pellets and supernatants (ESA) were analyzed. For each of the three independent biological replicates, the intensity of the ESA bands was assessed by band densitometry, and subsequent MIC2/GRA1 ratios are presented (no-IAA ratios are normalized). ****, *P* < 0.0001.

The dilated plasma membrane was examined by staining for the major surface antigen SAG1, and under both AC9- and ERK7-depleted conditions, the integrity of the membrane was compromised in some intracellular parasites (Fig. 4A). This leads to the apparent release of few micronemes outside the parasite body (Fig. 4B), as previously reported using EM in the absence of AC9 (1).

Although ERK7 and AC9 are essential for parasite survival, depleted mutants are not affected in intracellular growth and replication, as large vacuoles can be observed even after 48 h of IAA treatment (Fig. 4C). However, both mutants are seemingly able to lyse the parasitophorous vacuole (PV) membrane (PVM) but remain trapped inside the host cell even after the host cell detaches to form floating “bubbles” (Fig. 4C). In parallel, a more standard induced-egress assay highlights a severe defect in parasites lacking ERK7 (Fig. 4D; Fig. S4A). While the depletion of ERK7 was reported not to impact microneme secretion, a severe defect was observed in the absence of AC9 (1–3). The microneme secretion assay used by O'Shaughnessy et al. (3) was based on luciferase detection and applied previously to the CPH1-mAID strain (8). Here, induced secretion was monitored by the detection of MIC2 released into the supernatant (SN) upon cleavage, and protein was quantified by Western blotting. Under these conditions, ERK7-mAID-HA treated with IAA showed a complete block of microneme secretion, as reported for AC9 (Fig. 4E; Fig. S5A). Such a severe block in microneme secretion was also previously reported for AC10; TFP1, a protein essential for microneme biogenesis; as well as GC and UGO, two essential proteins involved in the signaling cascade leading to microneme exocytosis (1, 19–21). As a control, the CPH1-mAID strain was generated (Fig. S4B to D) and displayed a milder defect in microneme secretion, indicating that the discrepancy can be explained only in part by the type of assay used (Fig. S4E and Fig. S5B). Of note, in addition to the partial block of microneme secretion observed in CPH1-depleted parasites, the Western blot analysis also revealed that MIC2 is additionally processed, indicative of a defect in motility, as reported previously (22).

10.1128/mBio.02057-21.4 FIG S4 (A) Representative wide-field pictures of a typical induced-egress assay. IMC1 stains the pellicle of the parasites, while GRA1 stains the parasitophorous vacuole (intact or remnant after egress). Graphs in Fig. 4D are derived from such IFAs. Even after induction with BIPPO, ERK7-depleted parasites did not egress. (B) CPH1 was C-terminally tagged with the mAID-HA construct as shown by integration PCRs. (C) The CPH1-mAID-HA construct was tightly downregulated by IAA as verified by an IFA. Bars = 2 μm. (D) CPH1-depleted parasites failed to form lysis plaques after 7 days. TIR1 represents the parental strain. (E) Depletion of CPH1 caused a moderate defect in microneme secretion when stimulated with ethanol (EtOH). Anti-MIC2 antibodies were used for secretion (white arrow, full-length MIC2; black arrow, secreted MIC2) and anti-dense granule 1 (GRA1) for constitutive secretion; both pellets and supernatants (ESA) were analyzed. For each of the three independent biological replicates, the intensity of the ESA bands was assessed by band densitometry, and subsequent MIC2/GRA1 ratios are presented (no-IAA ratios are normalized) (*P* < 0.001). Download FIG S4, EPS file, 2.9 MB. Copyright © 2021 Dos Santos Pacheco et al. 2021 Dos Santos Pacheco et al. https://creativecommons.org/licenses/by/4.0/ This content is distributed under the terms of the Creative Commons Attribution 4.0 International license.

10.1128/mBio.02057-21.5 FIG S5 Two additional biological replicates for induced microneme secretion of the ERK7-mAID-HA (A) or the CPH1-mAID-HA (B) strain. The first biological replicates for each strain are presented in Fig. 4E and Fig. S4E in the supplemental material, respectively. (C) Analysis of total MIC2 levels by Western blotting shows the accumulation of MIC2 in ERK7- and AC9-inducible knockdown upon the addition of IAA. The accumulation is due to their inability to secrete their microneme content properly. GRA1 is used as a loading control. Quantification was performed by band densitometry. Download FIG S5, EPS file, 2.4 MB. Copyright © 2021 Dos Santos Pacheco et al. 2021 Dos Santos Pacheco et al. https://creativecommons.org/licenses/by/4.0/ This content is distributed under the terms of the Creative Commons Attribution 4.0 International license.

### Comparative proteomics of wild-type and ERK7/AC9-depleted parasites identify novel candidate components of the apical complex.

**FIG 5:**
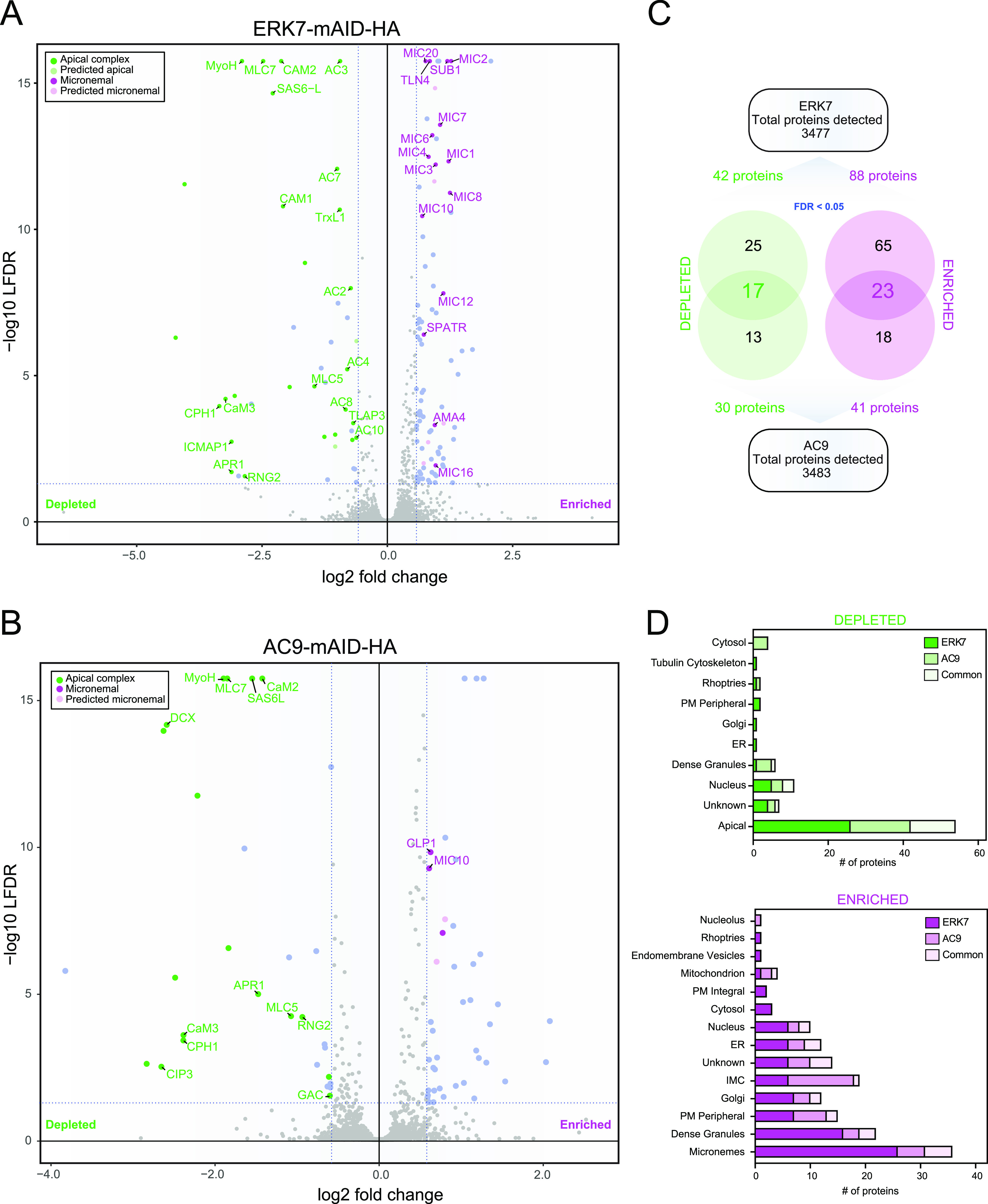
Comparative proteomics of ERK7- and AC9-depleted parasites. (A and B) Volcano plots showing all the proteins found in ERK7 (A) and AC9 (B) mAID parasites and their differential abundances in auxin-treated parasites versus untreated parasites (LFDR, local false discovery rate). The most significant changes are colored and grouped based on their known and predicted (LOPIT) localizations. On the left (in green) are proteins that are depleted and that majorly localize to the apical complex. The proteins on the right (in magenta) are the ones that are enriched and are found majorly within the micronemes. (C) Venn diagrams showing the significant changes in ERK7 and AC9 (proteins individually depleted or enriched) and the common proteins depleted or enriched in both ERK7 and AC9 auxin-treated parasites. (D) Predicted localization of enriched and depleted proteins in both ERK7 and AC9 according to hyperLOPIT.

**FIG 6:**
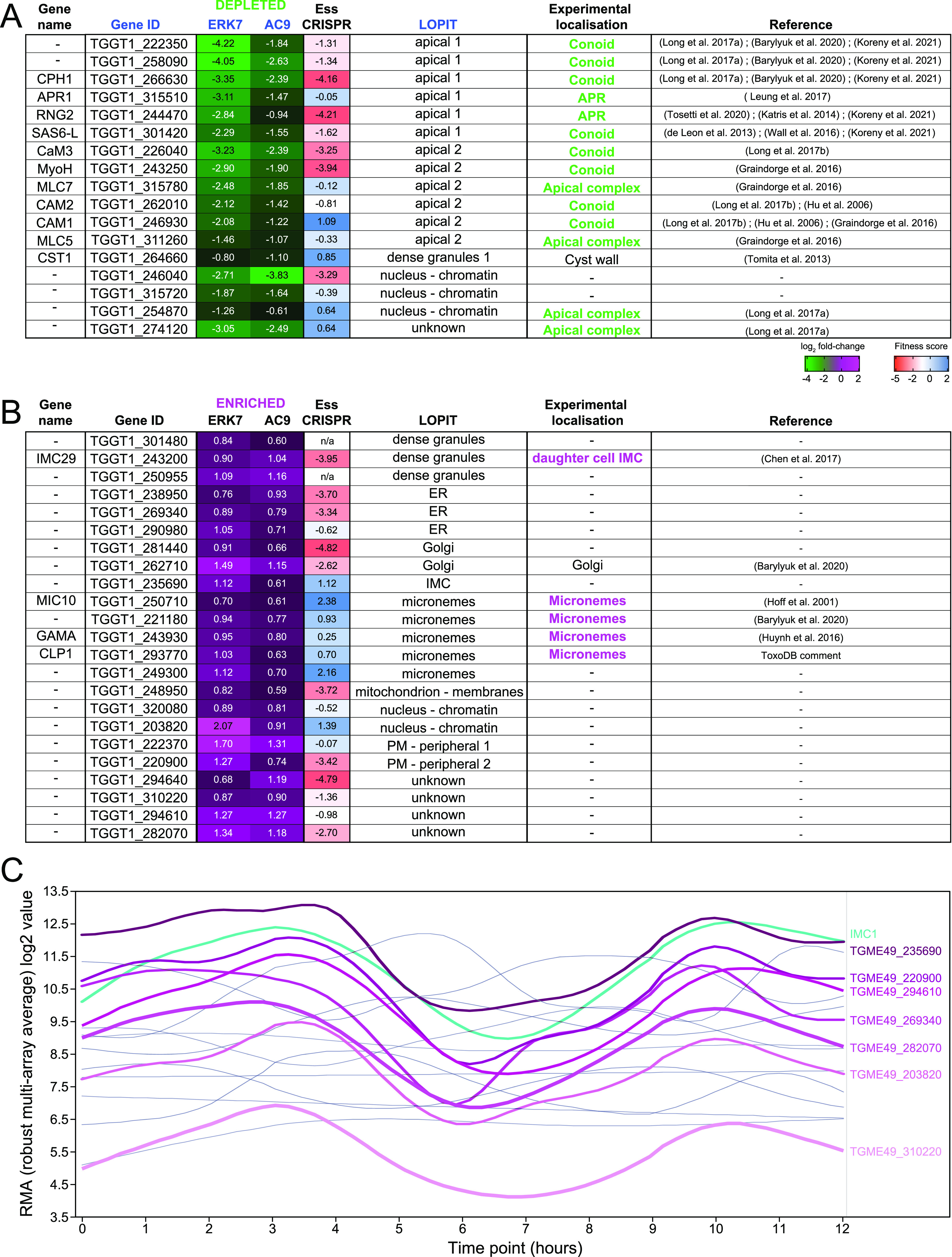
Statistically significant proteins depleted/enriched in both strains. (A and B) Heat maps showing the most significant changes (FDR < 0.05) under both ERK7 and AC9 (auxin-treated) conditions. In green (A) are the proteins that are significantly depleted, and in magenta (B) are the proteins that are significantly enriched in both ERK7 and AC9. Predicted (LOPIT) localizations and previously localized proteins are also indicated (1, 7, 8, 23, 24, 31, 40–47, 51). (C) Expression profile of proteins with no “experimental localization” presented in panel B. Out of 17 proteins, 7 of them (thick lines/shades of magenta) share a similar expression profile with a typical IMC-related protein, namely, IMC1 (cyan). The 10 others, with different expression profiles, are presented with thin gray lines. Data were retrieved from the ToxoDB website [Transcriptomic data “T. gondii ME49 Cell Cycle Expression Profiles (RH)” from reference 52].

Given the dramatic disappearance of the structure as well as the protein markers of the conoid and APR in both AC9- and ERK7-depleted parasites, we performed a comparative proteomics analysis by mass spectrometry (MS) to gain further knowledge about the composition of the apical complex. The data set analysis revealed a good overall coverage in comparison to the hyperplexed localization of organelle proteins by isotope tagging data set (hyperLOPIT) (3,832 detected T. gondii proteins) (23), with totals of 3,477 and 3,483 proteins detected in the ERK7 and AC9 samples, respectively (Fig. 5C). In agreement with previously published data and proteomics data sets, numerous known apical complex proteins disappeared in both AC9- and ERK7-depleted parasites (Fig. 5A, B, and D and Fig. 6A). Out of the 17 most significantly depleted proteins in both data sets, 14 were already localized at the apical pole of the parasite (Fig. 6A). Of relevance, the abundances of APR1 and RNG2, two markers of the APR (7, 24), are significantly reduced in both ERK7- and AC9-depleted parasites. Overall, the analysis points toward at least a dozen plausible novel candidate proteins potentially associated with the apical complex.

### Comparative proteomics of wild-type and ERK7/AC9-depleted parasites show an accumulation of microneme proteins and components of the IMC.

Besides the loss of several components of the apical complex, other distinct sets of proteins accumulate in ERK7- and AC9-depleted parasites (Fig. 5A, B, and D and Fig. 6B). In the absence of ERK7, known microneme proteins as well as proteins predicted by hyperLOPIT to be associated with micronemes (23) are significantly more abundant. This can be explained by the severe microneme exocytosis defect observed in the mutant parasites. Concordantly, the total amount of MIC2 was analyzed by Western blotting and accumulated in ERK7- and AC9-depleted parasites (Fig. S5C). In AC9-depleted parasites, microneme proteins also accumulated (Fig. S5C); however, other proteins predicted to be localized at the IMC or dense granules were also overrepresented. Out of the 17 proteins enriched in both data sets and not localized experimentally to date, 7 share a cell cycle transcription profile common to previously described IMC proteins (Fig. 6C). A comprehensive list of the comparative proteomics data for ERK7-mAID-HA and AC9-mAID-HA is presented in Data Set 1.

### Validation of new apical and putative inner membrane complex-associated proteins.

**FIG 7:**
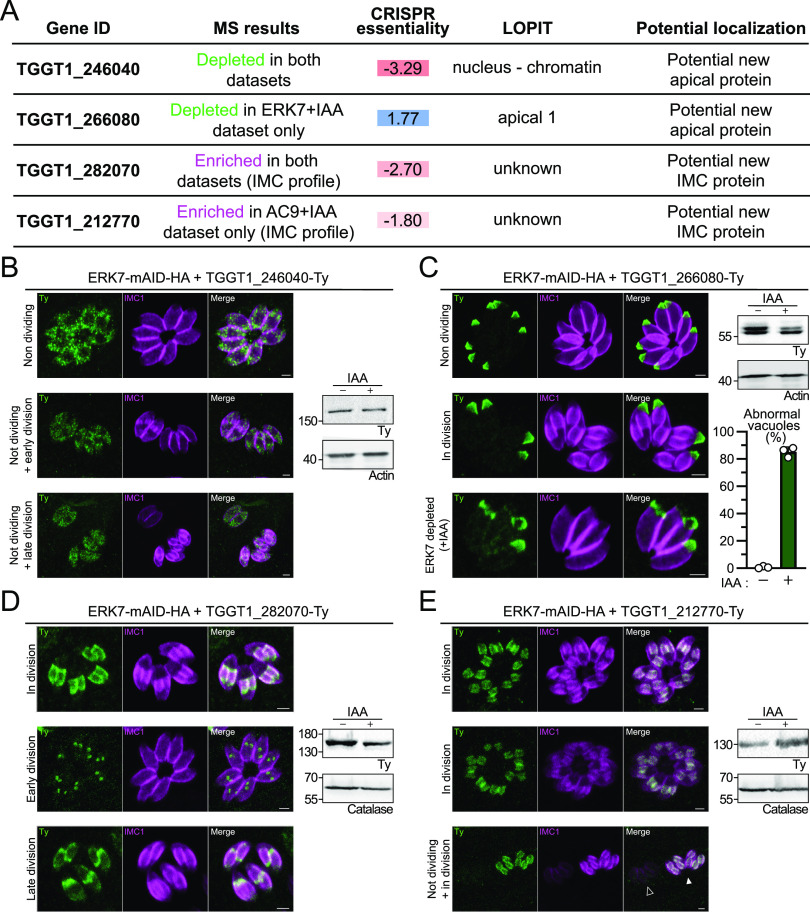
Validation of potential new apical and IMC proteins. (A) Table presenting the four candidates successfully tagged in the ERK7-mAID strain. (B) TGGT1_246040 localizes as discrete dots throughout the cytoplasm of nondividing and dividing parasites. Bars = 2 μm. (C) AC11 (TGGT1_266080) localizes only at the apical cap of mature cells and is affected by the disorganization of the apical pole in ERK7-depleted parasites. Bars = 2 μm. The graph presents the quantification of vacuoles with at least one parasite displaying abnormal AC11 staining. Around 200 vacuoles were counted for each of the three independent biological replicates. (D) IMC33 (TGGT1_282070) decorates the IMC of forming daughter cells. It appears early during daughter cell biogenesis and seems to accumulate at the growing end of the daughter cells before their emergence. Bars = 2 μm. (E) IMC34 (TGGT1_212770) decorates the IMC of forming daughter cells only. The black arrowhead indicates nondividing parasites with no IMC34 signal, while the white arrowhead indicates dividing parasites displaying clear IMC34 staining. Bars = 2 μm.

To validate the comparative proteomic data sets, four proteins of interest were tagged in the ERK7-mAID-HA strain (Fig. 7A). The protein TGGT1_246040 showed a dotty signal in the cytoplasm (despite being predicted to localize at the nucleus) (Fig. 7B) and a seemingly unaltered level of expression in the absence of ERK7. TGGT1_266080 was localized at the apical cap and was therefore named AC11 (Fig. 7C). Unlike the majority of apical cap proteins identified to date, AC11 is not detectable in nascent daughter cells. The level of AC11 is slightly decreased and the localization is perturbed in the absence of ERK7 (Fig. 7C). Both the TGGT1_282070 and TGGT1_212770 proteins localized at the IMC of forming daughter cells and were not detectable in mature parasites (Fig. 7D and E). Intriguingly, TGGT1_282070 appeared very early during parasite division, right at the start of daughter cell biogenesis (Fig. 7D). Also, toward the end of daughter cell formation, this protein was seemingly accumulating at the “growing edge” of the budding cells. Given the absence of predicted signal peptide or transmembrane-spanning domains, we tentatively assign them to the alveolin network and name them IMC33 and IMC34.

## DISCUSSION

AC9 is a component of the alveolin network proposed to serve as a regulatory platform for ERK7 kinase activity (2). In this study, we have revisited the role of ERK7 in the integrity of the apical complex of T. gondii in light of unmatching phenotypic consequences compared to AC9 depletion (1, 3). ERK7-depleted parasites phenocopied AC9 and AC10 depletion except that the APR was reported to be intact and microneme secretion was not affected. Here, a new ERK7-mAID-HA strain was generated and confirmed to be severely affected in invasion and egress from infected cells. U-ExM analysis proved that like AC9 and AC10, ERK7 is required to maintain conoid stability but also to preserve the tethering of the SPMTs in mature parasites. In contrast to a previous report, the depletion of ERK7 leads to the disappearance of the APR, as highlighted by EM images, as well as the degradation of APR1 and KinA and the mislocalization of RNG1. As a late marker of the APR, RNG1 might not be a structural component *per se* of the APR but rather is associated with the structure only in mature parasites. RNG1 appears to be expressed and stable in the absence of the APR but is mislocalized to the residual body in ERK7-depleted parasites. The absence of the APR explains the wide apical opening observed by EM and U-ExM and why individual microtubules are detached in deoxycholate extraction experiments. The APR is likely lost upon parasite final maturation, as it presumably serves as the MTOC for the SPMTs.

NHS-ester staining coupled with U-ExM offers novel opportunities to study in detail the internal structures of the parasite in its vacuolar niche. This would notably be the case for less intensely studied parasites such as *Cryptosporidium*, where a very small number of antibodies have been raised for localization studies (25). NHS-ester staining is of great value as it can reveal numerous structures such as the “ring structures” that presumably correspond to the micropores or the ring structures described with GAPM2 and GAPM3, two resident proteins of the IMC (26, 27). The basal complex of the parasite appears intensely stained, probably highlighting a concentration of protein complexes present at the basal pole of the parasite. In addition, very small structures in dividing parasites like the centrocone and centromeres are surprisingly evident with NHS-ester staining that only EM could reveal until now (28). Further improvements of these methods could potentially help retain membrane- or lipid-bound epitopes as well as reaching a greater expansion ratio without the need for heavy instruments (29).

The induced microneme secretion monitored here by excretory-secretory antigen (ESA) detection via Western blotting provides a radically opposing outcome for ERK7-mAID-HA compared to published results based on the luciferase assay. The presence of the conoid seems required for microneme secretion as no processed MIC2 can be detected in the supernatant from parasites depleted of ERK7, AC9, or AC10 (1). Since Long et al. used a very sensitive assay based on MIC2 fused to a luciferase reporter, we also generated a CPH1-mAID mutant previously analyzed by the same method to include and compare in the analysis (8). The two assays are rather concordant for CPH1-depleted parasites, although the mild defect in microneme secretion observed when analyzing ESA by Western blotting was not detected in the luciferase-based assay (8). The accumulation of additional MIC2-processed forms, visible only by the Western blot-based assay, reflects a defect in gliding motility. Under this circumstance, MIC2 is not translocated to the posterior and is trimmed by the apical subtilisin-like serine protease SUB1, as previously reported (22). It is plausible that the luciferase assay is too sensitive for the detection of minor defects of microneme exocytosis. Long et al. also looked indirectly at microneme secretion by monitoring mVenus diffusion from the PV to the host cell cytoplasm. This diffusion is indicative of permeabilization of the PVM by the action of the secreted microneme protein perforin PLP1 (30). Both AC9- and ERK7-depleted parasites are indeed able to lyse the PVM but remain trapped inside the host cell. This suggests that a low level of microneme exocytosis is occurring in a few intracellular parasites but is insufficient to trigger complete egress and hence results in parasites trapped in floating detached host cells. The discrepancies with regard to the loss of the APR and the block of microneme secretion between the two studies remain to be clarified.

Capitalizing on the loss of the APR and conoid that resulted in the degradation of several apical complex proteins in both ERK7- and AC9-depleted strains, we embarked on a proteomics analysis to compare these mutants to the nondepleted parasites and identify novel components of the affected structures. The comparative analysis between each replicate revealed a striking quantitative reproducibility of the number of peptides for the vast majority of proteins. Consequently, the subsets of proteins disappearing or accumulating in the samples emerged clearly. A large set of proteins already known or predicted to be associated with the apical complex were shown to disappear, while only a small number of new candidate apical complex proteins could be found. This might reflect a technical limitation of mass spectrometry in detecting small, low-abundance, or not easily digested proteins. Alternatively, the combination of approaches used so far to determine the repertoire of apical complex proteins might be almost exhaustive. Of relevance, known as well as predicted microneme proteins accumulated in ERK7- and AC9-depleted parasites as a consequence of the impairment of these mutants in microneme exocytosis. This tendency was more pronounced in the ERK7-depleted parasites since twice as many proteins were found significantly enriched in those parasites compared to the AC9-depleted ones (88 versus 41). This could be due to the intrinsic nature and role of the two proteins: while AC9 is a structural component of the alveolin network, ERK7 is an active kinase (2). Alternatively, the AC9-mAID-HA parasites were growing slower than ERK7-mAID-HA parasites, and hence, the majority of parasites were still intracellular and syringed out of the host cells at the time of harvesting. The large fraction of intracellular AC9-mAID-HA parasites were not triggered to secrete micronemes and distributed in all phases of the cell cycle, likely explaining the limited changes in microneme proteins and the increase in IMC proteins compared to those of ERK7-mAID-HA that were extracellular and mainly in G_1_ phase. Of note, the slower growth of AC9-mAID-HA than of ERK7-mAID-HA was IAA independent and turned out to be due to contamination with mycoplasma. Despite this shortcoming, the data on AC9-mAID-HA were included here as these parasites were used only as controls and actually led to the identification of two new IMC proteins, IMC33 and IMC34.

In the absence of ERK7 or AC9, some proteins predicted by hyperLOPIT to localize to the dense granules, endoplasmic reticulum (ER), Golgi apparatus, or nucleus also accumulated (23). The experimental confirmation of their localization deduced from LOPIT data is pertinent since proteins such as the product of TGGT1_254870, mainly predicted to localize at the nucleus, were previously localized at the apical pole of the parasite (8). Similarly, the protein TGGT1_243200, predicted to localize to the dense granules, is associated with the IMC of nascent daughter cells (31). Here, the endogenously epitope-tagged protein TGGT1_246040 predicted to localize to the nucleus is found dotted in the cytoplasm. IMC33 and IMC34, which were enriched after the depletion of AC9 and exhibited an IMC transcription profile, were found associated with the IMC of the nascent daughter cells but were undetectable in the mother cells. Finally, AC11 is a new apical cap protein of the mother but is undetectable in the daughter cells, which is in contrast to most AC proteins described to date. Besides scrutinizing their localization, we also assessed the down/upregulation of proteins in the depleted parasites to compare them with the quantitative proteomic data sets. The vast majority of the downregulated proteins indeed disappeared upon the depletion of ERK7, as they were part of the conoid complex. However, some proteins, like TGGT1_246040, seem not to be affected by ERK7 depletion. The same is true for IMC33, for which the protein levels seem stable, while an upregulation upon ERK7 depletion was observed in the comparative proteomics analysis. The discrepancy between the protein levels observed experimentally and the results of quantitative proteomics can, to a large extent, be explained by the timing of parasite harvesting following depletion and the proportion of intracellular versus extracellular parasites.

Overall, the role of ERK7 was characterized more deeply with U-ExM, NHS-ester staining, EM, and comparative proteomics. The absence of ERK7, a kinase that likely phosphorylates several components (known and unknown) of the apical complex, dislocates the conoid complex and the APR. The severe microneme secretion defect also strengthens previous observations that exocytosis of these organelles, and, as a consequence, proper egress, gliding motility, and invasion, requires the presence of an intact conoid.

## MATERIALS AND METHODS

### Generation of strains.

T. gondii parasites were grown in human foreskin fibroblasts (HFFs) (ATCC CRL-1634) in Dulbecco’s modified Eagle’s medium (DMEM; Gibco) supplemented with 5% fetal calf serum (FCS), 2 mM glutamine, and 25 mg/ml gentamicin. HFFs were obtained from the ATCC (ATCC CRL-2088, reference number CCD1072Sk). ERK7-mAID-HA (TGGT1_233010) was obtained by transfecting a guide RNA (gRNA) obtained by a Q5 site-directed mutagenesis kit (New England BioLabs) in the pSAG1::Cas9-U6::sgUPRT vector (32) with primer 5′-CACGCCGCATTTGTTGACTGGTTTTAGAGCTAGAAATAGC-3′ and KOD PCR with forward (F) primer 5′-TTTCAGTCTGCGTCCAAGACATACAACAGCGCTAGCAAGGGCTCGGG-3′ and reverse (R) primer 5′-CGGCTCTTCTTTGACACAAAGCAAGAGTCTATACGACTCACTATAGGG-3′, as described previously (33). Depletion of ERK7-mAID-HA was performed with 500 mM auxin (IAA) (34). Knock-ins of APR1, KinA, and RNG1 were performed as previously described (1). Cloning was performed in Escherichia coli XL-1 Gold chemically competent bacteria. Freshly egressed T. gondii tachyzoites were transfected by electroporation, and mycophenolic acid (25 mg/ml) and xanthine (50 mg/ml) (for the hypoxanthine-guanine phosphoribosyltransferase [HXGPRT] cassette) or pyrimethamine (1 mg/ml) (for the dihydrofolate reductase [DHFR] cassette) were employed to select resistant parasites.

### Immunofluorescence assay.

Parasites were grown in HFF cells with coverslips in 24-well plates for 24 to 30 h before fixation with either 4% paraformaldehyde (PFA)–0.05% glutaraldehyde (PFA/GA) or cold methanol and neutralized in 0.1 M glycine–phosphate-buffered saline (PBS) for 5 min. For SAG1 staining, parasites were permeabilized with 0.1% saponin instead of Triton X-100, which was used for all other IFAs. The following antibodies were used: rabbit anti-IMC1 (35); rabbit anti-hemagglutinin (HA) (Sigma); mouse anti-acetylated tubulin (clone 6-11B-1; Santa Cruz Biotechnology); mouse anti-ISP1 (36); rabbit anti-ARO (37); mouse anti-Ty, anti-MIC2, and anti-ROP2-4 (gifts from J.-F. Dubremetz, Montpellier); anti-tubulin AA344 scFv-S11B (β-tubulin) and AA345 scFv-F2C (α-tubulin); and the secondary antibodies Alexa Fluor 405-, Alexa Fluor 488-, and Alexa Fluor 594-conjugated goat anti-mouse/anti-rabbit. For Western blotting, secondary peroxidase-conjugated goat anti-rabbit or -mouse antibodies (Sigma) were used. Confocal images were obtained with a Zeiss laser scanning confocal microscope (LSM700, apochromat 63×/1.4 oil objective). Ultrastructure expansion microscopy (U-ExM) was performed as previously described (1), and images were taken with a Leica TCS SP8 stimulated emission depletion (STED) system with lightning deconvolution. For intracellular conditions, the parasites were seeded on nonconfluent host cells to guarantee the good expansion of the sample. All U-ExM gels were carefully measured to ensure a minimum expansion ratio of 4×. NHS-ester (DyLight 488 NHS-ester; Thermo Fisher) was used at 5 μg/ml and incubated for 1 h in PBS. Confocal and expansion images were processed with ImageJ and LAS X, respectively. Images were obtained at the Bioimaging Core Facility of the Faculty of Medicine at the University of Geneva.

### Electron microscopy.

For negative staining, extracellular or scrapped-syringed retrieved parasites, either grown or not in the presence of IAA, were pelleted in PBS. Conoid protrusion was induced by incubation with 40 μl of BIPPO (5-benzyl-3-isopropyl-1*H*-pyrazolo[4,3-*d*]pyrimidin-7(6*H*)-one) in PBS for 5 min at 37°C. Four microliters of the sample was applied onto a glow-discharged 200-mesh Cu electron microscopy grid for 10 min. The excess sample was removed by blotting with filter paper and immediately washed 3 times on drops of double-distilled water. Finally, the sample was negatively stained with a 0.5% aqueous solution of phosphotungstic acid (PTA) for 20 s and air dried. Electron micrographs of parasite apical poles were collected with a Tecnai 20 transmission electron microscope (FEI, Netherlands) operated at an 80-kV acceleration voltage and equipped with a side-mounted charge-coupled-device (CCD) camera (MegaView III; Olympus Imaging Systems) controlled by iTEM software (Olympus Imaging Systems).

### Plaque assay.

HFFs were inoculated with fresh parasites and grown for 7 days with or without IAA. HFFs were then fixed with PAF/GA, washed once with PBS, and stained with crystal violet.

### Microneme secretion.

After ∼48 h of growth, parasites were harvested and centrifuged at 1,000 × *g*. Pellets were then washed twice with intracellular buffer prewarmed at 37°C (5 mM NaCl, 142 mM KCl, 1 mM MgCl_2_, 2 mM EGTA, 5.6 mM glucose, and 25 mM HEPES [pH 7.2]). Next, parasites were incubated at 37°C for 15 min in DMEM containing 2% ethanol (EtOH). After induction, the parasites were centrifuged at 1,000 × *g* for 5 min at 4°C; the supernatants (SNs) were collected in different tubes and centrifuged at a higher speed (2,000 × *g*) for 5 min at 4°C to clean the parasite debris; and pellets were washed once in PBS. Pellets and SNs were analyzed using anti-MIC2, anti-catalase (CAT) (parasite lysis control), and anti-dense granule 1 (GRA1) (constitutive secretion control) antibodies by Western blotting.

### Parasite extraction with sodium deoxycholate.

Freshly egressed parasites (with or without IAA) were deposited on top of poly-l-lysine-coated coverslips and treated with 10 mM deoxycholate (20 min at room temperature). Parasites were fixed with cold methanol for 8 min, and IFAs were performed with anti-acetylated α-tubulin antibody.

### Induced-egress assay.

T. gondii tachyzoites were grown for 30 h on HFF cells with coverslips in 24-well plates. The infected host cells were incubated for 7 min at 37°C with DMEM containing BIPPO prior to fixation with PFA/GA. IFAs were performed as described above with anti-IMC1 and anti-GRA3 antibodies, and 200 vacuoles were counted. Data are presented as mean values ± standard deviations (SD) (three independent experiments).

### Mass spectrometry.

**(i) Sample preparation.** Parasite pellets were resuspended in 100 μl of 1% RapiGest surfactant (Waters) in 0.1 M triethylammonium bicarbonate (TEAB) and 7.5 mM dithioerythritol (DTE). Samples were heated for 5 min at 95°C. Lysis was performed by sonication (6 times for 30 s) at 70% amplitude and with a 0.5-s pulse. Samples were kept for 30 s on ice between each cycle of sonication. Samples were centrifuged for 5 min at 16,000 × *g*. The protein concentration was measured by a Bradford assay (38), and 50 μg of each sample was subjected to protein digestion as follows. The sample volume was adjusted to 100 μl with 0.1 M TEAB to obtain a final concentration of RapiGest of 0.1%. Two microliters of 50 mM DTE in distilled water was added, and reduction was carried out at 60°C for 1 h. Alkylation was performed by adding 2 μl of iodoacetamide (400 mM in distilled water) for 1 h at room temperature in the dark. Overnight digestion was performed at 37°C with 5 μl of freshly prepared trypsin (Promega) (0.2 μg/μl in 0.1 M TEAB). To remove RapiGest, samples were acidified with trifluoroacetic acid (TFA), heated at 37°C for 45 min, and centrifuged for 10 min at 14,000 × *g*. The supernatants were then desalted with a C_18_ microspin column (Harvard Apparatus, Holliston, MA, USA) according to the manufacturer’s instructions, completely dried under a speed vacuum, and stored at −20°C.

**(ii) ESI-LC-MS/MS.** Samples were diluted in 50 μl of loading buffer (5% CH_3_CN, 0.1% formic acid [FA]), and 2 μl was injected onto the column. Electrospray ionization liquid chromatography-tandem mass spectrometry (ESI-LC-MS/MS) was performed on an Orbitrap Fusion Lumos Tribrid mass spectrometer (Thermo Fisher Scientific) equipped with an Easy nLC1200 liquid chromatography system (Thermo Fisher Scientific). Peptides were trapped on an Acclaim pepmap100 C_18_, 3-μm, 75-μm by 20-mm nano trap column (Thermo Fisher Scientific) and separated on a 75-μm by 500-mm C_18_ ReproSil-Pur (Dr. Maisch GmbH) 1.9-μm, 100-Å homemade column. Analytical separation was run for 180 min using gradients of 99.9% H_2_O–0.1% FA (solvent A) and 99.9% CH_3_CN–0.1% FA (solvent B). The gradient was run from 5% solvent B to 28% B in 160 min, then to 40% B in 20 min, and then to 95% B in 10 min, with a final stay of 20 min at 95% B. The flow rate was 250 nl/min, and the total run time was 210 min. Data-dependent analysis (DDA) was performed with an MS1 full scan at a resolution of 120,000 FWHM (full width at half maximum), followed by as many subsequent MS2 scans on selected precursors as possible within the 3-s maximum cycle time.

**(iii) Database search.** Peak lists (MGF file format) were generated from raw data using the MS Convert conversion tool from ProteoWizard. The peak list files were searched against the Toxoplasma gondii GT1 database (ToxoDB) (release 48; 8,460 entries) combined with an in-house database of common contaminants using Mascot (version 2.5.1; Matrix Science, London, UK). Trypsin was selected as the enzyme, with one potential missed cleavage. The precursor ion tolerance was set to 10 ppm, and the fragment ion tolerance was set to 0.02 Da. Variable amino acid modifications were oxidized methionine and deaminated (NQ). The fixed amino acid modification was carbamidomethyl cysteine. The Mascot searches were validated using Scaffold 4.11.1 (Proteome Software). Peptide identifications were accepted if they could be established at >91.0% probability to achieve a false discovery rate (FDR) of <0.1% by the Scaffold Local FDR algorithm. Protein identifications were accepted if they could be established at >96.0% probability to achieve an FDR of <1.0% and contained at least 2 identified peptides. Protein probabilities were assigned by the Protein Prophet algorithm (39). Proteins that contained similar peptides and could not be differentiated based on MS/MS analysis alone were grouped to satisfy the principles of parsimony.

**(iv) Comparative proteomics: bioinformatics analysis.** For the comparative proteomics analysis, the average from three replicates (without and with auxin [IAA]) was used to compute the FDR. A cutoff of 0.05 for the FDR was applied to obtain the most significant hits. For the volcano plots, the log_10_ FDR (*y* axis) and the log_2_ fold change (log_2_FC), comparing parasites without and with auxin treatment (*x* axis), were computed. A cutoff of 0.5 was applied to the log_2_FC to mark significant changes. The plots were generated using R statistical software, and a heat map with common significant changes (depleted and enriched proteins) for both ERK7 and AC9 plus auxin was generated using GraphPad Prism v9. All the raw data can be found in Data Set S1 in the supplemental material.

10.1128/mBio.02057-21.6 DATA SET S1 Complete comparative proteomic data set (fold changes and protein abundances). Download Data Set S1, XLSX file, 0.4 MB. Copyright © 2021 Dos Santos Pacheco et al. 2021 Dos Santos Pacheco et al. https://creativecommons.org/licenses/by/4.0/ This content is distributed under the terms of the Creative Commons Attribution 4.0 International license.
